# The mechanism of *MYB* transcriptional regulation by MLL-AF9 oncoprotein

**DOI:** 10.1038/s41598-019-56426-7

**Published:** 2019-12-27

**Authors:** Lu Cao, Partha Mitra, Thomas J. Gonda

**Affiliations:** 1 School of Pharmacy, University of Queensland, Brisbane, QLD Australia; 2 Faculty of Medicine, The University of Queensland, Brisbane, QLD Australia; 3 Gallipoli Medical Research Institute, Greenslopes Private Hospital, Brisbane, QLD Australia; 4 Institute of Health and Biomedical Innovation, Queensland University of Technology, TRI, Woolloongabba, QLD Australia; 5 University of South Australia Cancer Research Institute, Adelaide, SA Australia

**Keywords:** Acute myeloid leukaemia, Transcription

## Abstract

Acute leukaemias express high levels of *MYB* which are required for the initiation and maintenance of the disease. Inhibition of *MYB* expression or activity has been shown to suppress MLL-fusion oncoprotein-induced acute myeloid leukaemias (AML), which are among the most aggressive forms of AML, and indeed *MYB* transcription has been reported to be regulated by the MLL-AF9 oncoprotein. This highlights the importance of understanding the mechanism of *MYB* transcriptional regulation in these leukaemias. Here we have demonstrated that the MLL-AF9 fusion protein regulates *MYB* transcription directly at the promoter region, in part by recruiting the transcriptional regulator kinase CDK9, and CDK9 inhibition effectively suppresses *MYB* expression as well as cell proliferation. However, *MYB* regulation by MLL-AF9 does not require H3K79 methylation mediated by the methyltransferase DOT1L, which has also been shown to be a key mediator of MLL-AF9 leukemogenicity. The identification of specific, essential and druggable transcriptional regulators may enable effective targeting of *MYB* expression, which in turn could potentially lead to new therapeutic approaches for acute myeloid leukaemia with MLL-AF9.

## Introduction

The *MYB* proto-oncogene encodes a transcription factor (MYB) that is a key regulator of haematopoiesis and leukaemogenesis (reviewed in^1,2^). It acts by blocking differentiation^3,4^ and promoting proliferation^5,6^ and cell survival^7,8^. High levels of *MYB* mRNA are found in acute myeloid leukaemia (AML) cells and reduced expression accompanies and is required for terminal differentiation^4,9,10^. Leukaemias generated by MLL-fusion proteins are one of the most aggressive haematopoietic cancer types. *MYB* expression is essential for the initiation and maintenance of MLL-fusion induced AML, and indeed these leukaemias are particularly sensitive to *MYB* inhibition when compared to normal haematopoietic cells and leukaemias induced by several other oncogenes^11–13^. These observations suggest that targeting *MYB* expression could be a promising approach for treating leukaemias with MLL-fusions, and in turn highlight the importance of understanding how *MYB* transcription is regulated in these leukaemias.

One reason for the hypersensitivity of MLL fusion-driven AML to *MYB* inhibition may be that *MYB* is a direct target of the fusion oncoprotein. Hess *et al*. found that MLL-ENL activated *Myb* indirectly through up-regulation of *Hoxa9* and *Meis1*^11^. Zuber *et al*. and Bernt *et al*. have reported binding of the MLL-AF9 oncoprotein to the *MYB* locus by ChIP-PCR and ChIP-seq^13,14^. However, the detailed mechanism by which MLL-fusions drive *MYB* gene transcription is not fully understood.

In several normal and cancer cell types, *MYB* expression is frequently regulated by a transcriptional elongation block imposed by a motif in the first intron, located 1.7 kb downstream of the transcription start site (TSS), which is comprised of a stem-loop (SL) forming sequence followed by a poly (dT) tract, designated the SL-dT motif in this report^15–17^. In estrogen receptor-positive (ER^+^) breast cancer cells, it has been shown that estrogen-ER recruits the positive transcriptional elongation factor-b (pTEFb) to phosphorylate, via its catalytic component CDK9, serine 2 of the C-terminal-domain (CTD) of Pol II to overcome the elongation block^17^ and allow *MYB* expression. Similarly, in other cell types such as colon cancer other factors including NF-kB may recruit pTEFb^18^.

This mechanism of *MYB* regulation is of potential relevance to MLL fusion-driven AML because several MLL fusion partners including AF9, ENL, AF4 and AF5q31, are members of the SEC/EAP multi-protein complex^19–21^ which also contains pTEFb. In addition to the CTD, pTEFb also phosphorylates and inactivates NELF and DSIF, two factors that induce Pol II to pause immediately after transcriptional initiation; collectively this leads to productive transcriptional elongation to generate full-length mRNA^22,23^. Furthermore, many MLL-fusion partners are reported to bind either directly or indirectly through intermediary proteins to the histone methyltransferase DOT1L^24–27^. The activity of DOT1L, the only known enzyme that catalyses methylation of Histone 3 Lysine 79 (H3K79me2) is tightly linked to actively transcribed chromatin^28^ and is required for the oncogenic activity of several MLL fusions^14,29^. Thus, while both CDK9 and DOT1L play important roles in gene transcription, the roles of these factors in *MYB* transcription directed by MLL-fusions have yet to be investigated. In the present report, we have confirmed binding of MLL-AF9 to the *MYB* promoter and subsequent stimulation of *MYB* transcription. We then studied the mechanism by which MLL-AF9 regulates *MYB* transcription, asking in particular whether it involves the intronic SL-dT motif and what the requirements for and roles of the key regulators CDK9 and DOT1L are.

## Results

### *MYB* transcription is stimulated by MLL-fusion oncoproteins

**Figure 1:**
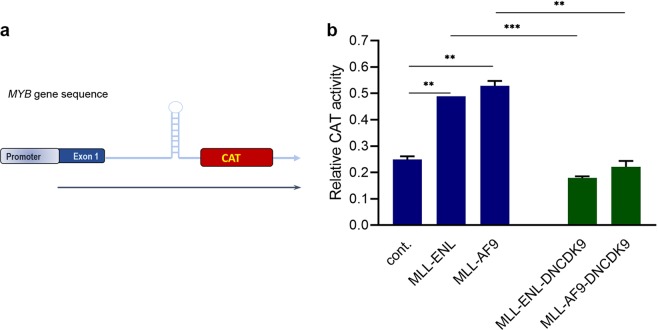
CAT reporter assays reveal that MLL fusions stimulate CDK9-dependent *MYB* transcription. (**a**) Schematic illustration of the MYB reporter construct containing the potential stem-loop-forming structure. (**b**) The reporter construct was co-transfected into HEK293T cells with an MLL-ENL or MLL-AF9 expression construct or an empty vector (blue bars). A dominant-negative CDK9 (DNCDK9) expression construct was co-transfected with these (green bars) as indicated. Asterisks indicate significant differences (*P ≤ 0.05, **P ≤ 0.005 and ***P ≤ 0.001) as determined by a two-tailed Student’s t-test.

To characterise the ability of MLL-fusions to directly stimulate the transcription of *MYB*, a construct which contains the *MYB* promoter, exon1 and approximately the first half of intron 1 (including the SL-dT motif) upstream of a CAT reporter gene was used^16^ (Fig. 1a). This construct was co-transfected into HEK293T cells with an MLL-ENL or MLL-AF9 expression construct or empty vector. Figure 1b shows that CAT activity increased 2-fold in the presence of MLL-ENL or MLL-AF9, indicating that both MLL fusions can stimulate *MYB* transcription. Next, to examine the role of CDK9 in this transcriptional stimulation, a dominant negative CDK9 (DNCDK9) construct, which carries a point mutation that functionally disables CDK9 catalytic activity, was co-transfected with the MLL-fusion and CAT constructs. Co-expression of DNCDK9 with the MLL-fusions prevented the stimulation of CAT activity. Thus, CDK9 activity appears essential for MLL-ENL and MLL-AF9 to activate *MYB* transcription from its promoter/SL-dT region.

### *Myb* expression is dependent on MLL-AF9 in an MLL-AF9-driven cell line

**Figure 2:**
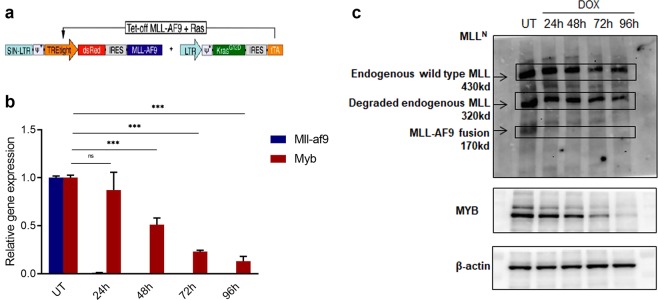
Correlation between the expression of MLL-AF9 and MYB in inducible MLL-AF9 leukaemia cells. (**a**) Retroviral vectors used to generate Tet-off MLL-AF9; Kras^G12D^ cell line. (**b**) RT-qPCR measurement of *MLL-AF9* and *Myb* expression upon treatment with DOX for the indicated times. Gene expression levels were normalized against *β-actin*. Asterisks indicate significant differences (ns, not significant, ***P ≤ 0.0001,) as determined by a two-tailed Student’s t-test. (**c**) Western blots to detect the expression of MLL, MLL-AF9 and MYB upon Dox treatment. β-actin was used as a loading control. The samples were run on 2 separate gels to detect MLL proteins and to detect MYB and β-actin, respectively; the blot of the latter was cut into higher and lower molecular weight regions and blotted with each antibody. Original blots are shown in Supplementary Information.

We next used an inducible, Tet-off MLL-AF9; Kras^G12D^ murine leukaemia cell line developed by Zuber *et al*.^13^; here it is referred to simply as the MLL-AF9 cell line. The expression of the MLL-AF9 gene and a dsRed tracer is dependent on a tetracycline-controlled transactivator protein (tTA) which is suppressed by doxycycline (DOX), while Kras^G12D^ and tTA transcription are controlled by a constitutive retroviral LTR promoter (Fig. 2a). When treated with 1 ug/ml DOX, cell proliferation was inhibited, and the morphology changed to that of more differentiated cells (Supplementary Fig. S1). *MLL-AF9* and *Myb* expression at the RNA and protein levels was measured by RT-qPCR and Western Blotting, respectively, following collection at 24, 48, 72 and 96 hours of DOX treatment. As shown in Fig. 2b, after DOX treatment *MLL-AF9* RNA almost disappears within 24 hours, while the effect on *Myb* is much slower, requiring 96 hours for a 90% reduction. The results of Western Blotting matched those at the RNA level, i.e. a rapid decrease of MLL-AF9 and a gradual decrease of MYB (Fig. 2c) were observed. (Note that the rabbit polyclonal antibody used, which recognises the N-terminal region of MLL, detected the 430 kd wild-type MLL protein, the 170 kd MLL-AF9 fusion protein encoded by the retroviral vector and a 320 kd N-terminal degradation product of wild-type MLL protein.) These results confirmed that MLL-AF9 drives *Myb* expression in this cell line, as reported previously^13^.

### MLL-AF9 oncoprotein regulates *Myb* transcription at the promoter region by binding to the promoter and recruiting CDK9

**Figure 3:**
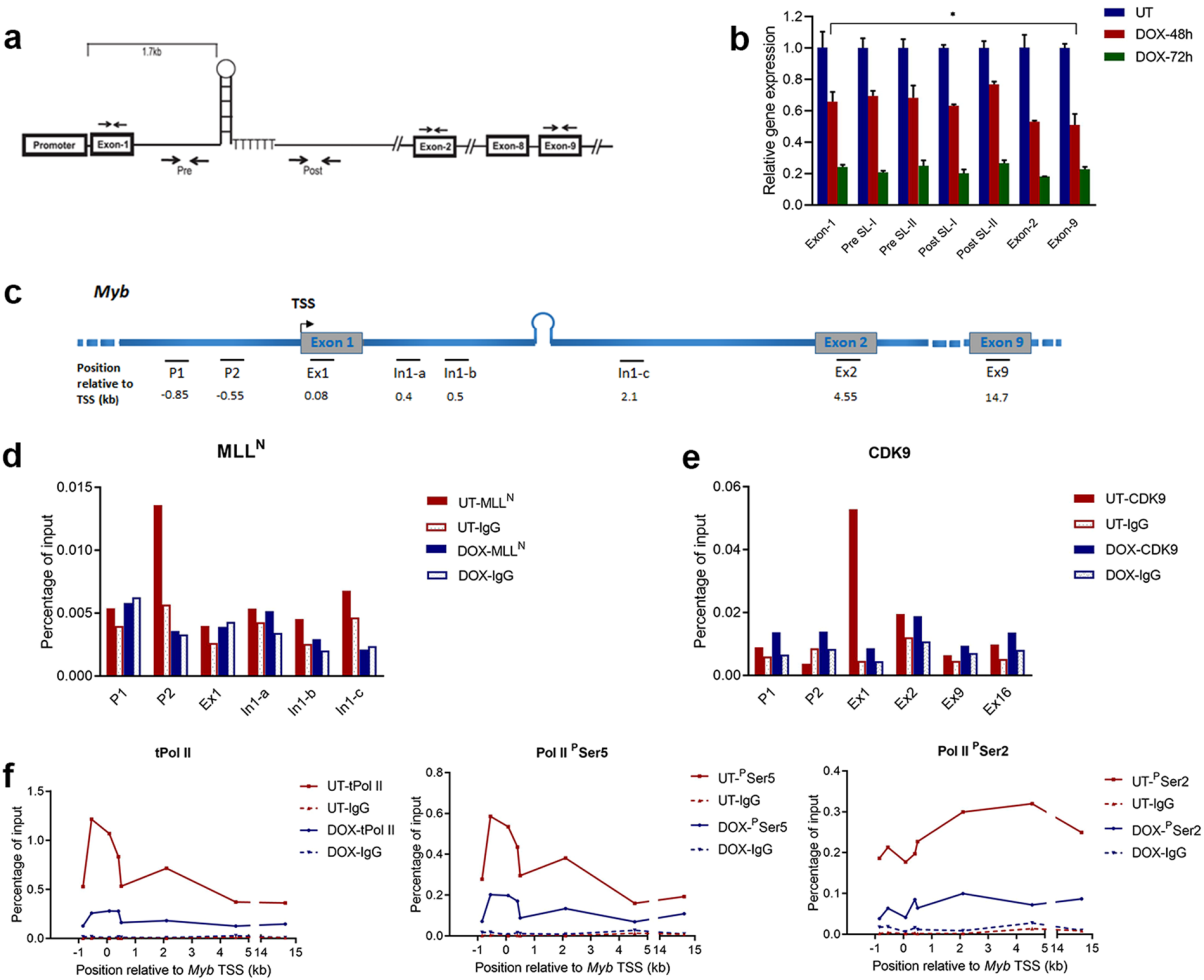
MLL-AF9 oncoprotein regulates *Myb* transcription at the promoter region by binding to the promoter and recruiting CDK9 (**a**) Positions of RT-qPCR primers used for detecting *Myb* gene transcription across the gene. (**b**) Exonic and intronic *Myb* transcription levels after MLL-AF9 was turned off by adding DOX. The expression levels were normalised against *β-actin* and plotted in comparison to untreated samples. Using a two-tailed Student’s t-test, comparing to Exon-1, there were no significant (*P* < 0.05) differences in the levels of any other transcripts at either 48 h or 72 h DOX treatment, (except for *P = 0.0251 between Exon-1 and Exon-9 at 48 h DOX treatment). (**c**) Positions of ChIP primers on *Myb* gene. Horizontal black lines (**—**) indicate primers; the values underneath are the positions relative to the *Myb* TSS (in kb). (**d,e**) MLL-AF9 cells were incubated with or without Dox for 72 h and then collected for ChIP assay with MLL^N^ (**d**) or CDK9 (**e**) antibody, IgG as a negative control. The amount of enrichment on *Myb* was measure by qPCR using primers along the gene. (**f**) MLL-AF9 cells were incubated with or without DOX for 72 h and then collected for ChIP assay with total Pol II (left), Pol II ^P^Ser5 (middle) and Pol II ^P^Ser2 (right) antibodies respectively, or with IgG. UT: untreated MLL-AF9 cells; DOX: MLL-AF9 cells incubated with DOX for 72 h; tPol II: total Pol II; ^P^Ser5: phosphorylation at Ser5 of the Pol II CTD; ^P^Ser2: phosphorylation at Ser2 of the Pol II CTD.

To determine whether *Myb* transcriptional elongation in the MLL-AF9 cells is blocked at the SL-dT motif, as reported in ER^+^ breast cancer and colon cancer, we used intronic RT-qPCR primers corresponding to sequences before and after the SL-dT region (pre-SL and post-SL), as well as exonic RT-qPCR primers representing exons 1, 2 and 9 (refs. ^17,30^), as depicted in Fig. 3a. The region where *Myb* transcriptional regulation occurs was investigated by comparing the levels of transcripts across these locations near and downstream of the TSS. As shown in Fig. 3b, after switching off MLL-AF9 expression with 48- or 72-hour DOX treatments, transcription of all of these regions, including exon 1, was reduced at similar rates and to similar extents, indicating that transcriptional control by MLL-AF9 acts on the promoter rather than within the first intron as observed for ER in breast cancer^17,30^.

Next, to confirm that MLL-AF9 directly binds to *Myb* and identify the binding site, ChIP assays were performed using the MLL antibody in untreated and 72-hour DOX-treated MLL-AF9 cells (in which MLL-AF9 was turned off completely and *Myb* expression was reduced by 80%). Primers upstream of the TSS and along the transcribed region of the *Myb* gene were used to detect MLL-AF9 binding (Fig. 3c). Figure 3d shows that an MLL-AF9 binding peak was detected at around 500 bp upstream of the *Myb* TSS in the untreated group (with primer pair P2), when *MLL-AF9* transcription remained turned on, but as expected, no binding above control levels was found in the DOX-treated group (when *MLL-AF9* was shut off). The result provides further evidence that *Myb* transcriptional activation requires the recruitment of MLL-AF9 at/near the promoter and that the *Myb* gene is a direct target gene of MLL-AF9.

Because our reporter assay study (Fig. 1) implied that MLL fusions can directly activate transcription from the *MYB* promoter/Intron 1 region in a CDK9-dependent manner, and because several such fusions including MLL-AF9 recruit pTEFb/CDK9^19,25^, we next examined CDK9 recruitment using ChIP. CDK9 showed a peak at the exon 1/TSS region in the untreated group (in which MLL-AF9 was expressed and *Myb* was abundantly expressed), while no peak was seen in the DOX-treated group where MLL-AF9 was absent and *Myb* expression was very low (Fig. 3e). This suggests that that CDK9 is recruited by MLL-AF9 to the *Myb* promoter and plays an essential role in *Myb* transcription, consistent with our CAT reporter assay results.

Next, to examine the activity of MLL-AF9-recruited CDK9 and Pol II dynamics in *Myb* transcription more generally, the distribution of total and phosphorylated Pol II along the gene was measured. Pol II accumulated around the promoter region and was detected all along the gene, consistent with active transcription of the *Myb* gene, and decreased substantially in the DOX-treated group (Fig. 3f, left). In both the control and the DOX-treated groups, Pol II ^P^Ser5, which is associated with transcriptional initiation^31^ was enriched at the promoter region and peaked at approximately 500 bp upstream of TSS (Fig. 3f, middle). In contrast, Pol II ^P^Ser2, indicative of elongation competence, was present at the *Myb* promoter and at increased levels downstream along the length of the *Myb* gene (Fig. 3f right). Further, compared to the untreated group, both Pol II ^P^Ser5 and Pol II ^P^Ser2 were dramatically decreased in the DOX-treated group. This is consistent with a reduced level of *Myb* gene transcription. Overall, the distributions of total Pol II and phosphorylated Pol II are consistent with the changes in *Myb* transcriptional levels. These distributions suggest that MLL-AF9 controls *Myb* transcription by recruiting CDK9 to the promoter region, which then phosphorylates Ser 2 of Pol II to promote transcriptional elongation.

### CDK9 inhibitors act mainly at the promoter to suppress *MYB* transcription in MLL-AF9 leukaemia cells

Since we showed above that CDK9 is essential for MLL-AF9- and MLL-ENL-driven *MYB* transcription in reporter assays and that it is recruited to *MYB* by the MLL-AF9 protein, we wished to investigate the effect of CDK9 inhibitors on *MYB* transcription, particularly in MLL-fusion driven leukaemia cells. First, we tested the inhibitory activity of the CDK9 inhibitors AT7519^32,33^ and BE-09-LN53 (referred to as i-CDK9 in ref. ^34^) on a panel of AML cell lines with (MOLM-13, MV4–11 and MLL-AF9 murine leukaemia) or without (HL-60 and U937) MLL rearrangements, since CDK9 inhibition was expected to affect expression of *MYB* and several other genes important for cell growth/viability. Supplementary Fig. S2a shows that both CDK9 inhibitors suppressed the growth/viability of all the human AML lines, with MOLM13, which expresses an MLL-AF9 fusion, being especially sensitive. Murine MLL-AF9 leukaemia cells were also highly sensitive to BE-09-LN53 but surprisingly, were barely affected by AT7519, even at nearly 4 times the concentration required to completely kill MOLM13 cells (Supplementary Fig. S2b).

**Figure 4:**
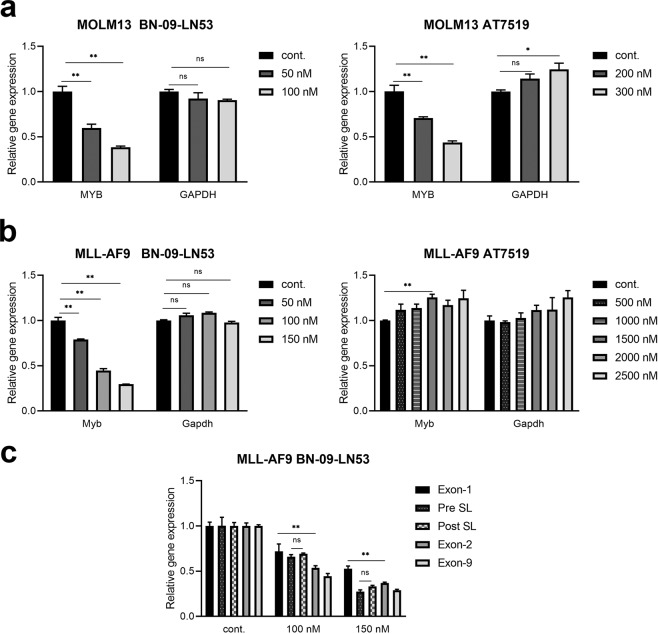
CDK9 inhibitors downregulate *MYB* transcription primarily at the promoter in MLL-AF9 leukaemia cells. **(a,b**) Human MOLM13 cells (**a**) or mouse MLL-AF9 cells (**b**) were incubated with indicated concentrations of CDK9 inhibitors BE-09-LN53 or AT7519 for 4 h. *MYB* transcription levels were measured by RT-qPCR using primers spanning *MYB* exon 8/9. Gene expression was normalised against *β-actin*. (**c**) MLL-AF9 cells were treated with BN-09-LN53 for 4 h and RT-qPCR was performed using the primers depicted in Fig. 3a. Relative expression of *Myb* intronic and exonic transcripts was determined using the level of *β-actin* as an internal control. Asterisks indicate significant differences (*P ≤ 0.001, **P ≤ 0.0001, ns, not significant) as determined by a two-tailed Student’s t-test.

Next, *MYB* mRNA levels were measured in both MOLM13 and murine MLL-AF9 leukaemia cells following treatment with each CDK9 inhibitor. Figure 4a,b show that both AT7519 and BE-09-LN53 rapidly (by 4 hours) reduced *MYB* expression in MOLM13 cells, while *Myb* expression in the murine MLL-AF9 cells was similarly suppressed by BE-09-LN53 but not by AT7519. To further examine on-target activity of the inhibitors, we assessed Pol II phosphorylation by Western Blotting in MOLM13 and MLL-AF9 leukaemia cells. As expected, AT7519 selectively diminished the phosphorylation of Ser 2, but not Ser 5, residues of Pol II CTD in MOLM13 cells (Supplementary Fig. S3a). However, in the murine MLL-AF9 cell line, Pol II phosphorylation was not affected by AT7519 at concentrations that completely inhibited phosphorylation of Pol II in MOLM13 cells. When the drug concentration was increased to an extremely high dose of 2000 nM, levels of not only Ser 2, but also Ser 5, phosphorylation decreased, at the same time, the amount of total Pol II was unchanged (Supplementary Fig. S3b). In contrast, BN-09-LN53 potently and specifically inhibited Ser 2 phosphorylation in these cells (Supplementary Fig. S3c). Thus, the activities of these two CDK9 inhibitors in suppressing CTD Ser 2 phosphorylation were consistent with the effects on both cell viability and *MYB* expression. These results further imply that AT7519 is inactive on murine MLL-AF9 leukaemia cells due to drug-specific properties (possibly uptake or species-specificity) rather than any difference in requirements for CDK9 activity *per se*.

In view of the findings above showing the importance of CDK9 and its recruitment by MLL-AF9 in the regulation of *MYB* expression, we next investigated how and where within the *MYB* gene CDK9 inhibition was acting. In particular we asked whether the mechanism in MLL-AF9 leukaemia cells was the same as that we reported in breast and colon cancers, in which transcriptional elongation is blocked at the SL-dT motif in the first intron^15–18^ by CDK9 inhibitors. Accordingly, we used the previously-described^17^ RT-PCR primer pairs (Fig. 3a) to detect exonic and intronic *Myb* transcripts upstream and downstream of the SL-dT region, and primers from exon 9 to measure mature transcript levels. As seen in Fig. 4c, transcription across exons 1 and 2, and the levels of mature transcripts, all decreased upon BN-09-LN53 treatment of MLL-AF9 cells. While there was a small increase in the ratio of exon 1 to exon 2 transcripts (1.3 to 1.4-fold), importantly there was no selective suppression of transcription downstream, as compared to upstream, of the SL-dT region. These data show that the downregulation of *Myb* by CDK9 inhibition mainly occurred at the promoter region, with possibly a small degree of pausing within the first intron, but not by an elongation block at the SL-dT region as described in solid cancer cell types^15–18,30^.

### DOT1L is not a key factor in regulating *MYB* transcription

In recent years, there has been a focus on the role of the protein methyltransferase DOT1L in MLL-fusion-transformed leukaemias. This is due to its involvement in elevating transcription of leukaemia-relevant genes, such as *MEIS* and *HOXA9*, which is achieved by its interaction with MLL-fusion oncoproteins and the resultant epigenetic modification of such genes by H3K79 methylation^14,35–39^. In particular, the interaction between DOT1L and MLL-AF9/ENL has been confirmed by identifying a 10-amino acid region of DOT1L essential for binding to MLL-AF9^40^. Since we have demonstrated that *Myb* expression is regulated directly by MLL-AF9 and is dependent on CDK9 activity, we were interested in determining whether regulation of *Myb* by MLL-AF9 is also reliant on DOT1L activity/H3K79 methylation.

**Figure 5:**
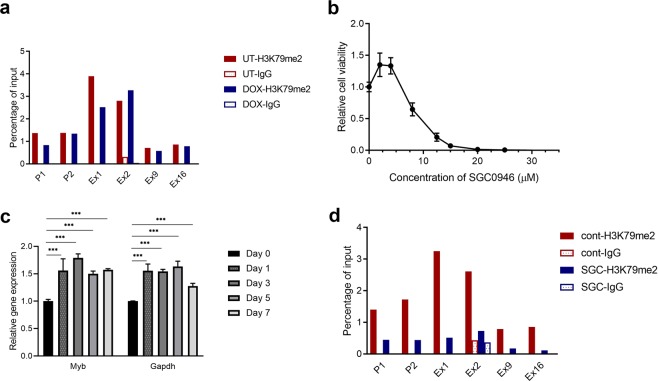
The role of H3K79 methylation in *MYB* transcriptional regulation in the MLL-AF9 cell line. (**a**) MLL-AF9 cells were cultured with or without DOX for 72 h and collected for ChIP assays using H3K79me2 antibodies respectively, or corresponding IgG as a negative control, followed by qPCR using primers depicted in Fig. 3c. Signals were normalised to that of the input. (**b**) MLL-AF9 cells were treated with increasing concentrations of SGC0946 or DMSO solvent for 48 h and then subjected to a Resazurin assay to determine cell viability. Experiments were done in triplicate. Error bars indicate SD and averages were plotted relative to DMSO-treated controls. (**c**) MLL-AF9 cells were incubated with 5 μM of SGC0946 or DMSO solvent for the indicated times. *Myb* mRNA levels were measured by RT-qPCR using primers for *Myb* exon 9 and normalised against *β-actin* mRNA (***P ≤ 0.001 by a two-tailed Students t-test). (**d**) MLL-AF9 cells were treated with 5 μM of SGC0946 or DMSO solvent for 3 days. ChIP assays were performed using an anti-H3K79me2 antibody or corresponding IgG, followed by qPCR using the indicated primers (see Fig. 3c). Signals were normalised to that of the input. UT: untreated; DOX: 72 h DOX treatment.

To this end, ChIP assays were undertaken using an antibody against H3K79me2, the modification generated by DOT1L, to indicate DOT1L activity. As shown in Fig. 5a, high levels of H3K79me2 were detected along the *Myb* gene in untreated and 72-hour DOX-treated cells, and these were particularly high at the promoter regions. In the DOX-treated cells, where MLL-AF9 was no longer expressed and *Myb* expression was very low, H3K79me2 levels barely changed compared to the untreated group. Surprisingly, the presence of H3K79me2 was not correlated with either the expression of *MLL-AF9* or *Myb*. That the disappearance of MLL-AF9 did not reduce H3K79me2 levels implies that in addition to MLL-AF9, which has been reported to recruit DOT1L^24,25^, it is likely that other proteins can also recruit DOT1L. It is also possible that even in the absence of DOT1L, the methyl groups remained on H3K79 due to the slow proliferation rate of the differentiated cells and thus slow turnover of histones bound to DNA.

To address whether *Myb* gene expression is subject to the control of DOT1L, a DOT1L antagonist SGC0946^41^ was used. SGC0946 treatment of MLL-AF9 cells resulted in extensive loss of cell viability (IC50 ~ 7.5 μM; Fig. 5b), in agreement with previous reports^29^. Next, MLL-AF9 cells were treated with 5 μM SGC0946 for up to 7 days and *Myb* mRNA levels were assessed. Interestingly, the *Myb* expression was not reduced after the addition of SGC0946. On the contrary, it resulted in increased expression of both *Myb* and the “housekeeping gene” *Gapdh* (Fig. 5c). To confirm that the dose and treatment time were sufficient to suppress or abrogate DOT1L activity, the presence of H3K79me2 across the *Myb* gene was examined by ChIP assays following SGC0946 treatment. It is apparent that 5 μM SGC0946 substantially diminished the degree of H3K79 methylation compared to the control after three days incubation (Fig. 5d). Thus, DOT1L activity is not necessary for MLL-AF9-induced *Myb* transcription in this leukaemia cell line.

## Discussion

The *MYB* gene and its activity are critical for leukaemic transformation^42–46^, and represents a potential therapeutic target in this disease (reviewed in ref. ^47^). Recent studies have shown that the MYB protein plays an essential role in MLL-AF9-induced AML transformation.^13^ and that this requires MYB’s interaction with CBP/p300^48–50^. Genome-wide ChIP-seq.^14^ and other ChIP studies (e.g. ref. ^13^) have shown that *MYB* is a target of the MLL-AF9 oncoprotein. Herein, we have provided evidence that MLL-AF9 can directly stimulate *MYB* transcription using reporter assays and by confirming that MLL-AF9 regulates *Myb* expression in murine AML cell line carrying a conditionally active MLL-AF9 expression vector. We further confirmed that MLL-AF9 occupies the *Myb* promoter region in this cell line, with a peak of binding ~ 0.5 kbp upstream of the TSS. Interestingly, while MLL-AF9 expression dropped rapidly in response to DOX treatment of these cells, downregulation of *Myb* expression was substantially slower, requiring 4 days to drop to 10% of the original level (even though the half-life of *Myb* mRNA is less than 2 hours^51^). This suggests that apart from MLL-AF9 as the major ‘driver’ of *Myb* transcription in these leukaemias, other factors contribute to *Myb* transcription; these may include factors that normally control *Myb* expression in progenitor-like cells. That CDK9 inhibition rapidly switches off *Myb* expression suggests that the normal, ie MLL-AF9-independent, regulation of *Myb* expression also requires CDK9 activity.

Having confirmed that *MYB* is a direct target of MLL-AF9, we examined the mechanism of *MYB* regulation and the roles of known, key mediators of the transcriptional regulatory activity of MLL-fusions. As mentioned in the Introduction, it is well established that in many systems *MYB* expression is regulated by a transcriptional elongation block within the first intron^15–18^. Furthermore, overcoming this block to allow continued transcription requires CDK9 activity and the resultant phosphorylation of the Pol II CTD at Ser2 residues^17,30^. Several MLL fusions are known to recruit complexes containing pTEFb, and thus its catalytic component, CDK9^21,25,52^, resulting in aberrant expression of a set of leukaemia-related genes^26^. Thus, we first examined the role of CDK9 activity in MLL-fusion-mediated regulation of *MYB*. Using both a dominant-negative form of CDK9 in reporter assays, and treatment of cell lines with pharmacological CDK9 inhibitors, it was found that CDK9 activity was indeed essential for *MYB* expression driven by MLL fusions.

We then asked whether *MYB* regulation by MLL-fusions involved overcoming an elongation block in the first intron and specifically, at the SL-dT motif required for this block in several other cell systems, as discussed above^15–18^. We did this through two approaches: examining the levels and distribution of Pol II and its key CTD-phosphorylated forms across the *Myb* gene in the DOX-regulated MLL-AF9 cell line, and measuring transcription across various regions along the length of the gene. The results showed that regulation of *Myb* expression by MLL-AF9 occurred primarily at/near the promoter rather than anywhere within the first intron. In particular, we did not observe an accumulation of Pol II at the SL-dT motif or anywhere else within the first intron when MLL-AF9 expression was switched off by DOX treatment. Furthermore, and most importantly, the levels of transcripts from exon 1 and from *all* downstream regions across the *Myb* gene decreased to the same extent after switching off MLL-AF9. This is in complete contrast to other systems where inhibition of *MYB* expression is accompanied by continued transcription upstream but not downstream of the elongation block/SL-dT region.

Thus, MLL-AF9 controls *MYB* expression predominantly by regulating transcription at the promoter region. This contrasts to the observations in breast and colon cancers discussed above, and in differentiation of other leukaemia cell lines^9,51,53^ in which *MYB* expression is controlled by transcriptional pausing in the first intron. The apparent lack of pausing in *Myb* intron 1 in MLL-AF9 cells suggests that factors that normally block elongation in this region may be deficient in these cells. Collectively, our and other observations suggest that *MYB* is regulated in different circumstances and cell types by different mechanisms and by different DNA-binding factors under each specific condition. Such factors include MLL-AF9 protein in MLL-AF9-transformed AML, estrogen receptor in breast cancer, and NF-kappa B in leukaemic differentiation^54^ and colon cancer cells^18^.

Another key effector of MLL fusions is the DOT1L histone methyltransferase. We found that the DOT1L inhibition impairs the proliferation of MLL-AF9 leukaemia cells, as previously reported^29,41^. However, even though the level of H3K79 di-methylation at the *Myb* locus was diminished dramatically after DOT1L inhibition, *Myb* mRNA expression remained high, indicating that the *Myb* transcriptional activation is not primarily dependent on DOT1L. Interestingly, Garcia-Cuellar *et al*.^55^ recently reported another form of MLL fusion protein MLL-ENL has two distinct clades of downstream targets: one group is dependent on DOT1L-mediated histone methylation for gene transcription, while the other group relies on pTEFb-mediated phosphorylation of RNA Pol II. This coincides with our finding in the context of *Myb* transcriptional regulation by MLL-AF9, which relies on CDK9 rather than DOT1L activity. Together, these observations suggest that DOT1L and CDK9 may exist in distinct complexes which function separately to regulate gene transcription^56^.

Overall, this study provides evidence that *MYB* is a direct target of MLL-AF9, and *MYB* transcriptional expression is largely dependent on CDK9 activity. CDK9 is presumably recruited to *MYB* by AF9 and other frequently-occurring MLL-fusion partners which have been identified as incorporated into macromolecular complexes that facilitate transcriptional elongation^21,25,52^. We suggest that the continued presence of CDK9, recruited by the MLL fusion, maintains high levels of *MYB* transcription from its promoter under conditions where transcription would normally be gradually turned off, as seen, for example, during differentiation of many myeloid progenitor-like cells. Interestingly, we also saw such a gradual decrease in *Myb* expression in MLL-AF9 leukaemia cells following the rapid switch-off of MLL-AF9 expression by DOX treatment. Finally, our findings reinforce, and provide a mechanistic basis for, the potential utility of CDK9 inhibitors for therapy of MLL-fusion-induced leukaemia^57,58^.

## Methods and Materials

### Cell culture

The murine Tet-off MLL-AF9 leukaemia cell line was obtained from Prof Ricky Johnstone (Peter MacCallum Cancer Centre, Melbourne, Australia)^13^. Cells were cultured in DMEM containing 10% FBS, supplemented with 0.55 mM L-Arginine HCl, 0.34 mM L-Asparagine, 0.0136 mM folic acid and 0.055 mM 2-Mercaptoethanol. Cells were passaged every 2–3 days to maintain a density between 2 × 10^5^~1 × 10^6^ cells/ml. To induce the shut-off of MLL-AF9 transcription, 1 μg/ml doxycycline was added into complete medium. U937, HL60, MOLM13 and MV411 human AML cells were maintained as suspension cultures in RPMI 1640 medium with 10% FBS in a 37 °C incubator with 5% CO_2_. Cultures were split 1:8–1:10 every two days to maintain a cell density between 5 × 10^4^~1 × 10^6^/ml.

### Chloramphenicol acetyltransferase (CAT) reporter assays

HEK293T cells were plated in 6-well plates (2 × 10^5^ cells/well) and incubated overnight, and then transfected with Lipofectamine 2000 with CAT reporter construct^16^, β-actin-promoter-driven luciferase expression vector, plus a combination of pBluescript, MLL-ENL/MLL-AF9 with/without DNCDK9 construct in serum and Pen/strep free Opti-MEM. After incubation for 4 hours, Opti-MEM was removed and changed to normal DMEM and then cells were incubated for a further 48 hours followed by harvesting for cell lysis. CAT expression was determined with a CAT ELISA kit (Roche, Basel, Switzerland) according to the specifications as recommended by the supplier. All assays were performed in triplicate. Optical density at 405 nm (OD405) was read using a microplate reader (iMark microplate reader, Bio-Rad, Hercules, CA, USA). CAT activity was normalized with respect to luciferase expression, which was determined by using LucLite (PerkinElmer, Waltham, MA) according to the protocol of the manufacturer.

### Cell viability assays

Triplicates of 5 × 10^3^ cells were seeded one day before the drug treatment in a 96-well flat-bottomed plate in a total volume of 100 μl per well in complete medium. 50 μl of serial diluted drug CDK9 inhibitor AT7519 (Selleck Chemicals, Houston, TX, USA) or BN-09-LN53 (Novartis, Basel, Switzerland) was added to each well. Triplicate blank wells were set up by adding the same volume of complete medium and diluted drug solvent DMSO. At the end of treatment, Resazurin was added to each well. After 2 hours’ incubation, absorbance was measured at ex544/em590 nM using FLUOstar Omega plate reader (BMG Labtech, Offenburg, Germany). The average absorbance values were calculated from triplicates and the average from blank wells was subtracted from all other absorbance values. Relative viability rates were calculated by normalizing to the rate of DMSO-treated cells and the error bars indicate standard deviation (SD).

### RNA extraction

Total RNA was extracted using PureLink® RNA Mini Kit (Ambion Life Technologies, Carlsbad, CA, USA) according to manufacturer’s instructions. The concentration of isolated RNA was determined using a spectrophotometer (NanoDrop ND-1000, Thermo scientific, Waltham, MA, USA). The ratio of absorbance 260 nm/280 nm and 260 nm/230 nm was used to assess the purity of RNA. A ratio of ~2.0 was accepted as sufficient for subsequent use.

### cDNA preparation

According the determined RNA concentrations, 1 μg RNA was taken from each sample. Prior to reverse transcription, all samples were treated with DNase I (Fermentas, Vilnius, Lithuania). RNA was converted into cDNA using the High Capacity cDNA Synthesis Kit (Applied Biosystems, Foster City, California, USA) following the temperature cycle: 25 °C for 10 min; 37 °C for 120 min; 85 °C for 10 min.

### RT-qPCR

Primers used for RT-PCR are listed in Supplementary Table 1. Reactions were set up with 300 nM forward primer, 300 nM reverse primer and 1x SYBR (Bioline, London, UK), except for reactions for detecting human *MYB* exon 1 which were made up with 1 μM forward primer, 1 μM reverse primer and 1x SYBR green. The conditions for measuring exonic transcripts were 95 °C for 3 min, and then 95 °C for 10 s, and 60 °C for 30 s for 40 cycles. The conditions for measuring intronic transcripts were 95 °C for 3 min, and then 95 °C for 10 s, 56 °C for 30 s and 72 °C for 30 s for 40 cycles. Data were analyzed using 2^−ΔΔCT^ relative quantitation method, normalized against exonic or intronic β-actin.

### Western blotting

To detect MYB and MLL-AF9 proteins, 100 µl SDS lysis buffer was added per 2 × 10^6^ cells. To detect total Pol II and phosphorylated Pol II, add 100 µl RAPI lysis buffer with protease inhibitor cocktail) was added per 2 × 10^6^ cells. Primary antibodies against MYB (Milipore 05–175), MLL-N (Bethyl A300–086A), β-actin (Santa Cruz Biotech sc-47778), Pol II (Cell Signaling Technology 2629), Pol II Phospho S5 (Cell Signaling Technology 13523), Pol II Phospho S2 (Cell Signaling Technology 13499) were used. Blots were developed using chemiluminescent reagent (Thermo Scientific) and the signals were detected with a ChemiDoc imaging system (Bio-Rad, Hercules, CA, USA).

### Chromatin immunoprecipitation (ChIP) assays

Cells were collected, cross-linked and lysed. Cells were sonicated using V1A Vibra-Cell Sonicator to achieve an average DNA fragment size of 500–1000 bp. An equal volume of Protein-A/G agarose beads were used to preclear the chromatin overnight at 4 °C before immunoprecipitation. In the meantime, the antibody MLL-N (Active Motif 39830), Pol II (Cell Signaling Technology 2629), Pol II Phospho S5 (Cell Signaling Technology 13523), Pol II Phospho S2 (Abcam ab5095), CDK9 (Bethyl A300–493A), H3K79 (Abcam ab3594), DOT1L (Bethyl A300–954A) was prebound overnight at 4 °C to Protein-A/G agarose beads (Santa Cruz Biotechnology). The next day, the precleared chromatin supernatant was equally divided and incubated with the protein-A/G agarose beads with prebound antibody on a rocker for 4 hours at 4 °C cold room. The beads were then washed and eluted to get the DNA-protein-antibody complexes. After reversing crosslinks DNA was purified using Invitrogen PCR purification kits in accordance with the manufacturer’s instructions. DNA levels were quantitatively measured by real time PCR and the data were represented as % of Input for each primer set. Primers used in the ChIP analysis are listed in Supplementary Table 2.

## Supplementary information

Suplementary Information

## Data Availability

All data generated or analysed during this study are included in this published article (and its Supplementary Information Files). No additional datasets were generated or analysed during this study. Reasonable requests for materials can be sent to the corresponding author.
