# Supplementary material for: The mechanism of MYB transcriptional regulation by MLL-AF9 oncoprotein
Source: Sci Rep. 2019 Dec 27;9:20084. doi: 10.1038/s41598-019-56426-7 (PMC6934848; doi:10.1038/s41598-019-56426-7)
Supplement: Supplementary file 1 — Suplementary Information [file 41598_2019_56426_MOESM1_ESM.pdf]

The mechanism of *MYB* transcriptional regulation by MLL-AF9 oncoprotein

Lu Cao, Partha Mitra, and Thomas J Gonda

SUPPLEMENTARY INFORMATION

Supplementary Figure S1. Characterisation of the inducible tet-off MLL-AF9-driven leukaemia cell line.

Supplementary Figure S2. Effect of CDK9 inhibitors on the viability of AML cell lines.

Supplementary Figure S3. Effect of CDK9 inhibitors on Pol II phosphorylation levels.

Supplementary Table S1. RT-qPCR primers

Supplementary Table S2. qPCR primers for ChIP assays

Original Blots for Figure 2C and Supplementary Figure S3 plus explanatory note (5 pages)

Supplementary Figure S1.

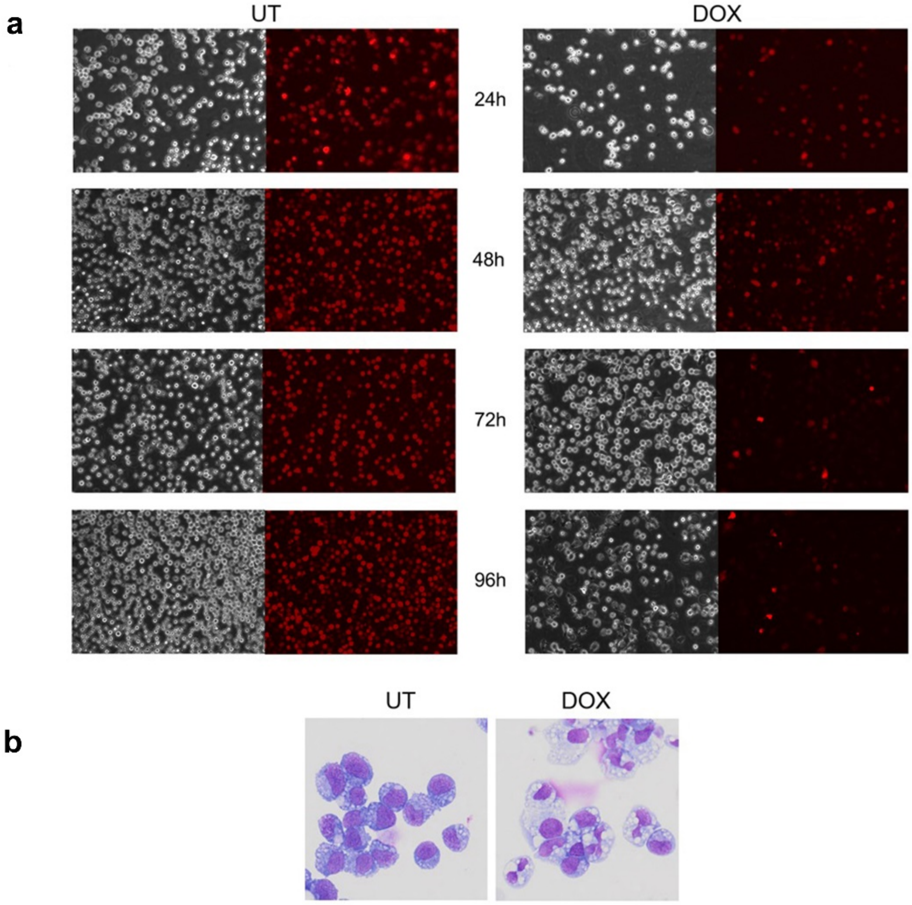

Supplementary Figure S1. Characterisation of the inducible tet-off MLL-AF9-driven leukaemia cell line.

a. Cells were incubated in the absence (left panel) or presence (right panel) of 1 μg/ml DOX for over 4 days and were observed under a fluorescence microscope. **b**. Cells were incubated without or with DOX for 72 h and were collected for cytospin followed by May-Grünwald-Giemsa staining.

Supplementary Figure S2.

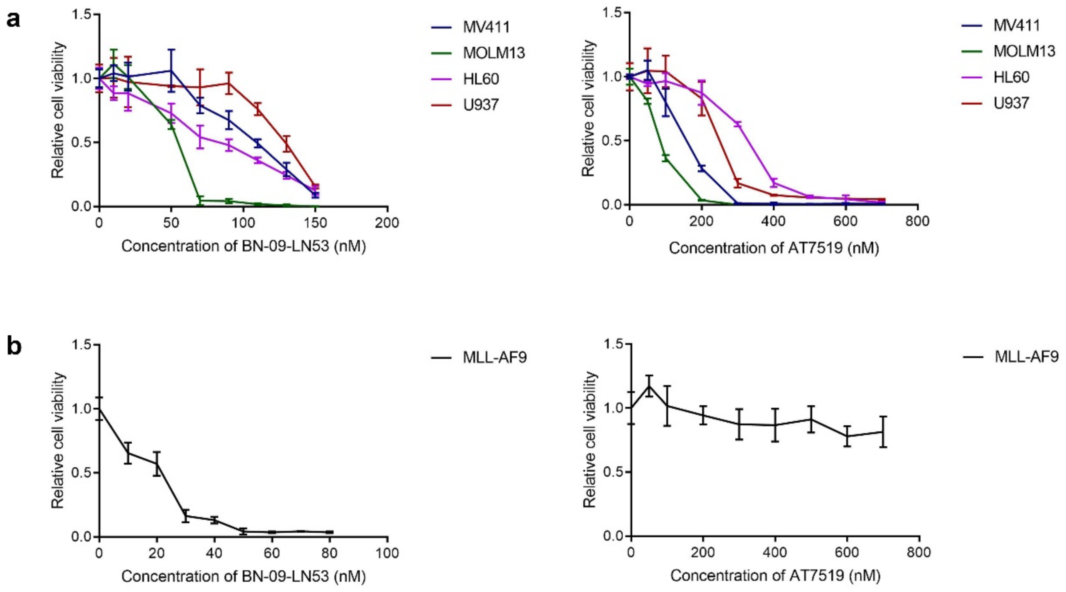

Supplementary Figure S2. Effect of CDK9 inhibitors on the viability of AML cell lines.

a. Human leukaemic cells were treated with increasing doses of CDK9 inhibitors BE-09-LN53 or AT7519 and then subjected to Resazurin assays to determine cell viability. **b.** Murine MLL-AF9 cells were treated with increasing doses of BE-09-LN53 or AT7519 and then subjected to Resazurin assays.

Supplementary Figure S3.

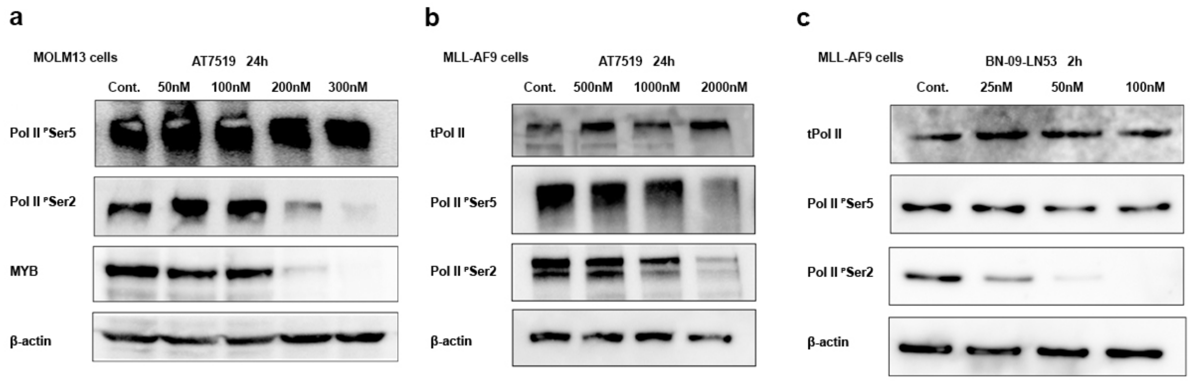

Supplementary Figure S3. Effect of CDK9 inhibitors on Pol II phosphorylation levels.

a. Human MOLM13 cells were treated with increasing concentrations of AT7519 for 24 h; **b.** Murine MLL-AF9 cells were treated with increasing concentrations of AT7519 for 24 h; **c.** Murine MLL-AF9 cells were treated with the indicated concentrations of BN-09-LN53 for 2 h. Pol II phosphorylation levels were detected by Western Blotting using phospho-serine 2 or phospho-serine 5 Pol II-specific antibodies, and MYB levels were detected using an anti-MYB antibody. β-actin was used as a loading control. tPol II: total Pol II; Pol II ^{P}Ser5: CTD serine 5-phosphorylated RNA polymerase II; Pol II ^{P}Ser2: CTD serine 2-phosphorylated RNA polymerase II.

Supplementary Table S1. RT-qPCR primers

Target	5’-3’ sequence
human *MYB* exon8/9-For	GCCAATTATCTCCCGAATCGA
human *MYB* exon8/9-Rev	ACCAACGTTTCGGACCGTA
human *MYB* exon1-For	CGTGACCTCCTCCTCCTCTT
human *MYB* exon1-Rev	CTGTGCCGGGGTCTTCG
human *MYB* exon2-For	TGACTATGATGGGCTGCTTC
human *MYB* exon2-Rev	TGTTTTCCCCAAGTGACGC
human *MYB* Pre-posing I-For	GAAATCCTCGTCCGAACTGTCA
human *MYB* Pre-posing I- Rev	GCGTGTGCTGCTGGGAAAG
human *MYB* Pre-posing II-For	CTCTGGGGACGAGAGGGCGACT
human *MYB* Pre-posing II-Rev	GCAGCACACCGTCCTGCG
human *MYB* Post-posing I-For	CCTCCGAATCACAGTAGC
human *MYB* Post-posing I-Rev	TTCTGTCAAGGAAACAAACC
human *MYB* Post-posing II-For	GTGGAGGCTAGACTAGAACC
human *MYB* Post-posing II-Rev	ACCCAGGAACAAGCAACC
human *GAPDH*-For	CGCTCTCTGCTCCTCCTGTT
human *GAPDH*-Rev	CCATGGTGTCTGAGCGATGT
human *β-actin*-For	AGAGCTACGAGCTGCCTGAC
human *β-actin*-Rev	AGCACTGTGTTGGCGTACAG
human *β-tubulin*-For	CTCTGAAGCTGACCACACCA
human *β-tubulin*-Rev	GCCAGGCATAAAGAAATGGA
Human *β-actin* Intron-For	TTGCTTTTTCCCAGATGAGC
Human *β-actin* Intron-Rev	GCTAAGTGTGCTGGGGTCTT
Human *MLL-AF9*-For	AATAAGCAGGAGAATGCAGG
Human *MLL-AF9*-Rev	TGCCTTGTCACATTCACCAT
mouse *Myb* exon9-For	AACCACACTTGCAGCTACCC
mouse *Myb* exon9-Rev	GCTGGTGAGGCACTTTCTTC
mouse *Myb* exon1-For	CCAAACCTCTTTGTTTGATGG
mouse *Myb* exon1-Rev	AGAAGGAGGTCACCGAGGAG
mouse *Myb* exon2-For	TGACTACGATGGGCTGCTGC
mouse *Myb* exon2-Rev	AGTTTTCCCCAAGTGACGCT
mouse *Myb* Pre-posing I-For	GCATTGAGGTGTGTTTGCAC
mouse *Myb* Pre-posing I-Rev	TCTTCAAACGGCTGGGTTTA
mouse *Myb* Pre-posing II-For	GCCGGGTTGTTTAGTAGCTG
mouse *Myb* Pre-posing II-Rev	TCTTCAAACGGCTGGGTTTA
mouse *Myb* Post-posing I-For	CGGGTCCTTTGTCGAAATTA
mouse *Myb* Post-posing I-Rev	TGTGCAAAGCCAAAGTCTTG

mouse *Myb* Post-posing II-For	GGCGACCAGGTCATTTTAGA
mouse *Myb* Post-posing II-Rev	AGTCTTGGGGTGAAATGTGG
mouse β-actin-For	*GATATCGCTGCGCTGGTCGT*
mouse *β-actin*-Rev	AGATCTTCTCCATGTCGTCC

Supplementary Table 2. qPCR primers for ChIP assays

**Target**	**5'-3' sequence**
ChIP-mouse *Myb* P1/-930--803 For	ACAGTCGAGCAATCCCAAGT
ChIP-mouse *Myb* P1/-930--803 Rev	TCCCAAACAAACAACCCCTA
ChIP-mouse *Myb* P2/-581--472 For	GCCACAGGGCTGACTGTATT
ChIP-mouse *Myb* P2/-581--472 Rev	CCCCCAACGATTTTTGACTA
ChIP-mouse *Myb* Ex1 For	CCAAACCTCTTTGTTTGATGG
ChIP-mouse *Myb* EX1 Rev	AGAAGGAGGTCACCGAGGAG
ChIP-mouse *Myb* In1-a/364-449 For	GCCTTCCCTGTTGGTTTGC
ChIP-mouse *Myb* In1-a/364-449 Rev	TGCGCTTCTCGCAGAGTT
ChIP-mouse *Myb* In1-b For	GCATTGAGGTGTGTTTGCAC
ChIP-mouse *Myb* In1-b Rev	TCTTCAAACGGCTGGGTTTA
ChIP-mouse *Myb* In1-c For	CGGGTCCTTTGTCGAAATTA
ChIP-mouse *Myb* In1-c Rev	TGTGCAAAGCCAAAGTCTTG
ChIP-mouse *Myb* Ex2 For	AGCGATGAAGATGATGAAGACA
ChIP-mouse *Myb* Ex2 Rev	CCCTTGTCCACCTAGTTTTCC
ChIP-mouse *Myb* Ex9 For	AACCACACTTGCAGCTACCC
ChIP-mouse *Myb* Ex9 Rev	GCTGGTGAGGCACTTTCTTC
ChIP-mouse *Myb* Ex16 For	GCTCGGAAATACGTGAACGC
ChIP-mouse *Myb* Ex16 Rev	TGGTGCTGCTCTCAACTGTT

Original Western blots for Figure 2C and Supplementary Figure S3 – Explanatory Note

The following images show the original Western blots used to in these Figures.

For **Figure 2C**, two separate gels were run *using the same amounts of the samples*. One (a 6% acrylamide gel) was blotted and probed with the MLL antibody to generate the top panel. The blot of the second (10% acrylamide) was cut to separate the higher and lower molecular mass regions which were then probed with MYB and β-actin antibodies respectively.

For **Supplementary Figure S3a**, three separate gels were run using the same amounts of the samples. Pol II ^{P}Ser 2 was blotted using an 8% acrylamide gel. To probe MYB and β-actin respectively a 10% acrylamide gel was used and the blot was cut at the 50 kD marker. A third 8% acrylamide gel was blotted and probed Pol II ^{P}Ser 5.

For **Supplementary Figures 3b** and **S3c**, one blot from an 8% acrylamide gel was cut to probe Pol II ^{P}Ser 2 and β-actin separately, and was then probed for tPol II following stripping of the Pol II ^{P}Ser 2 membrane. Pol II ^{P}Ser 5 was probed using a separate replicate blot.

Unfortunately, the files for the β-actin loading controls for Figures S3b and S3c were either missing or corrupted and so could not be shown here.

Figure 2C

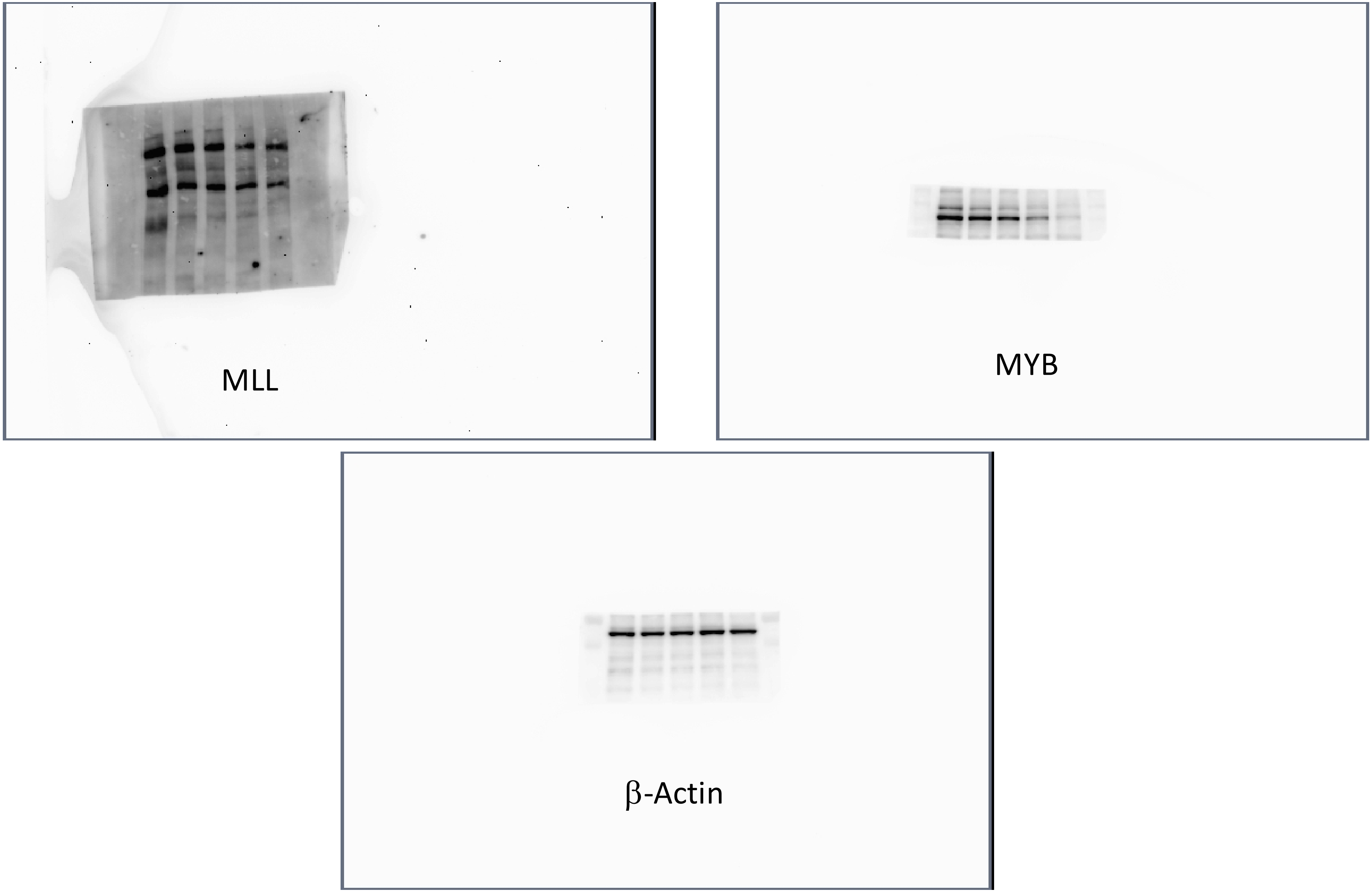

Supplementary Figure 3a

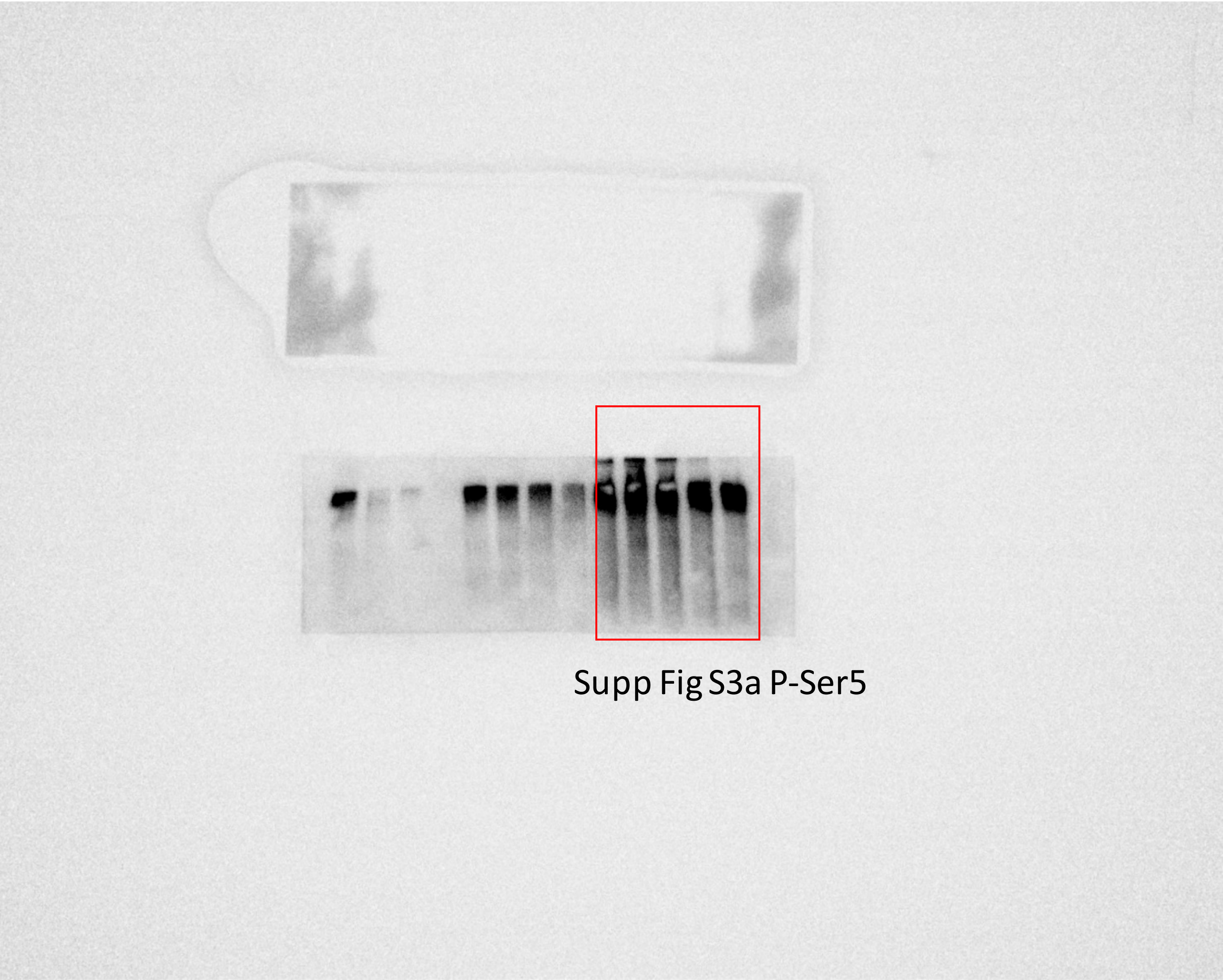

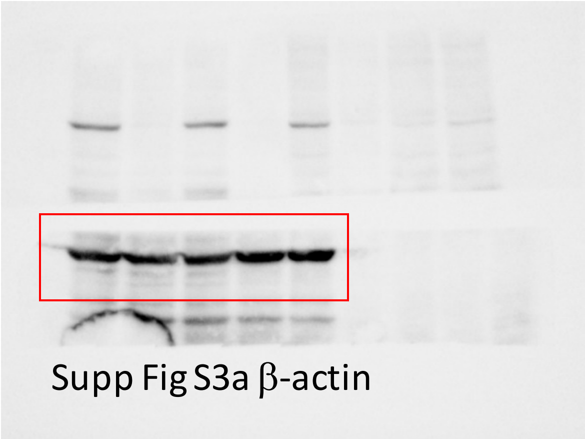

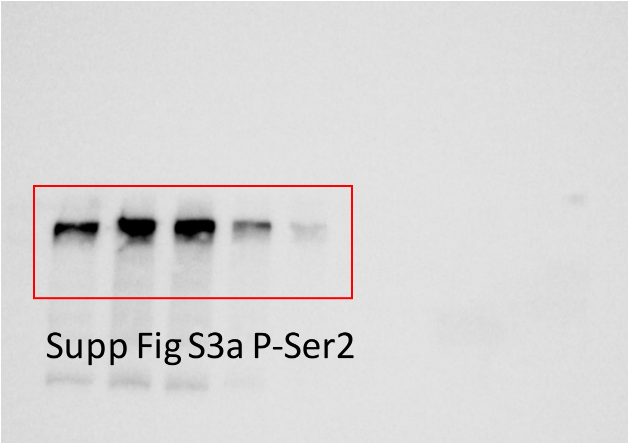

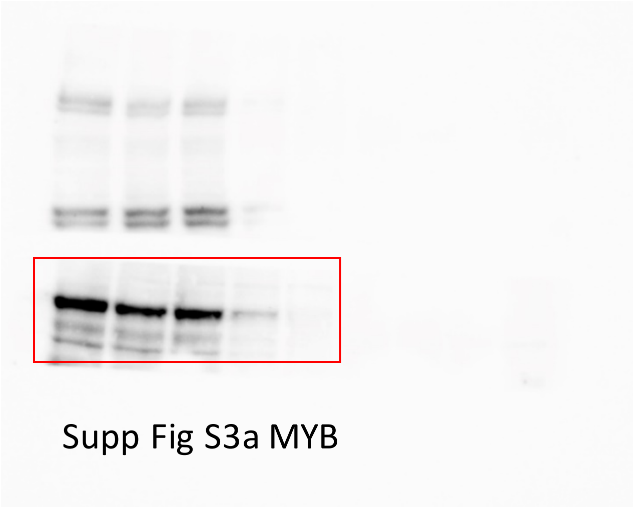

Supplementary Figure 3b

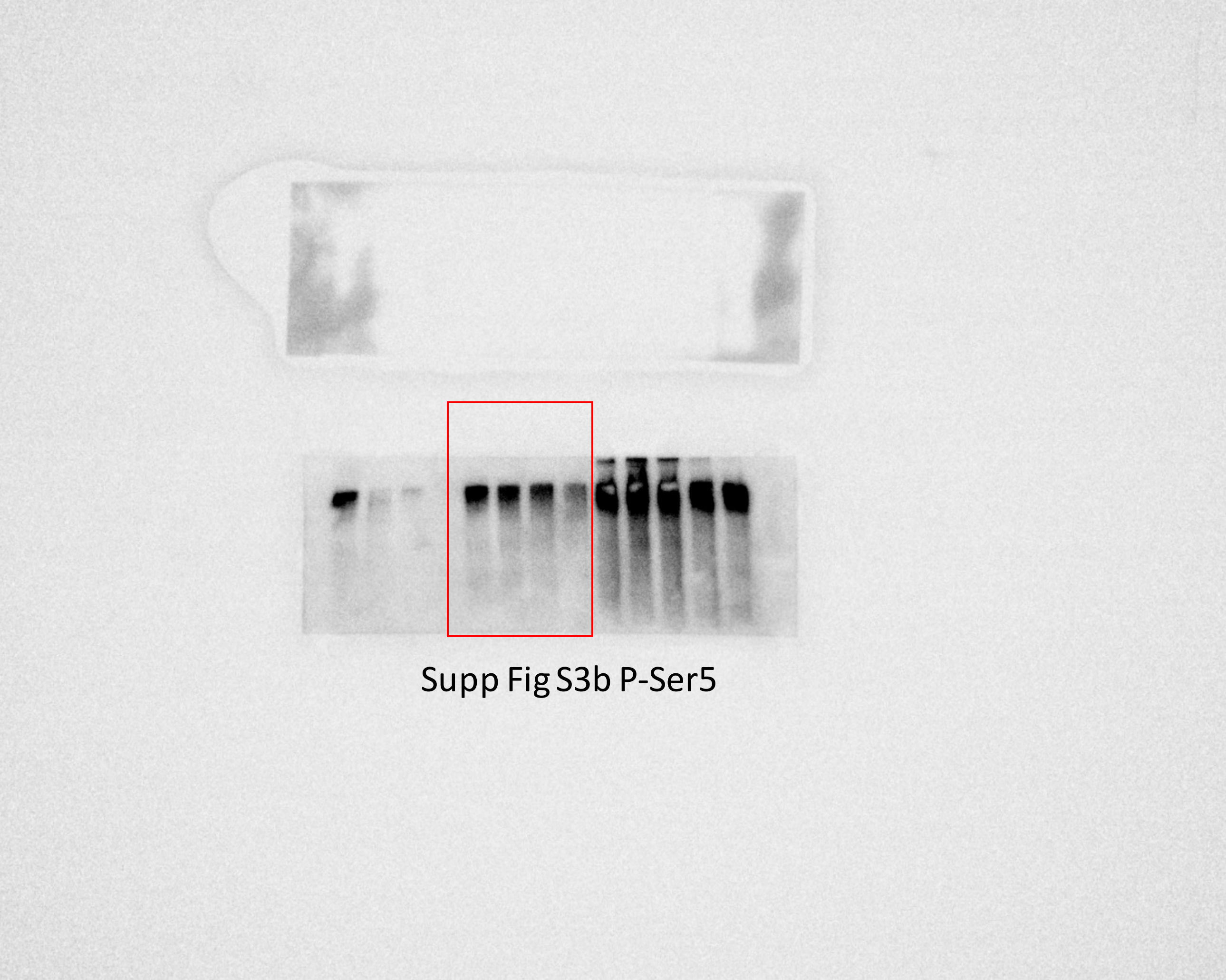

Supp Fig S3b P-Ser5

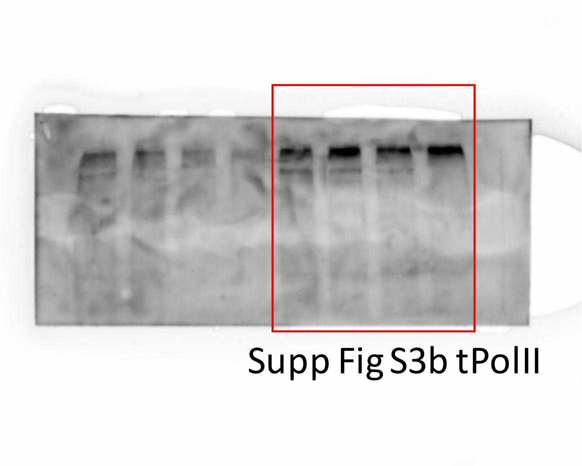

Supp Fig S3b tPolII

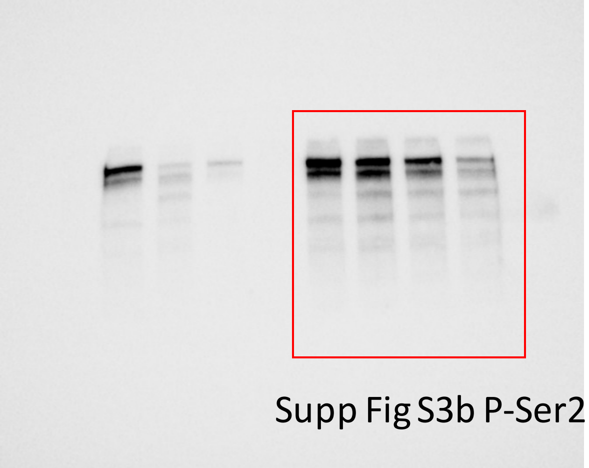

Supp Fig S3b P-Ser2

Supplementary Figure S3c

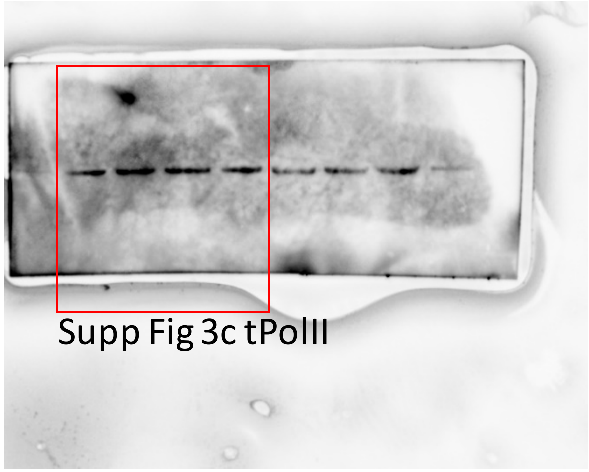

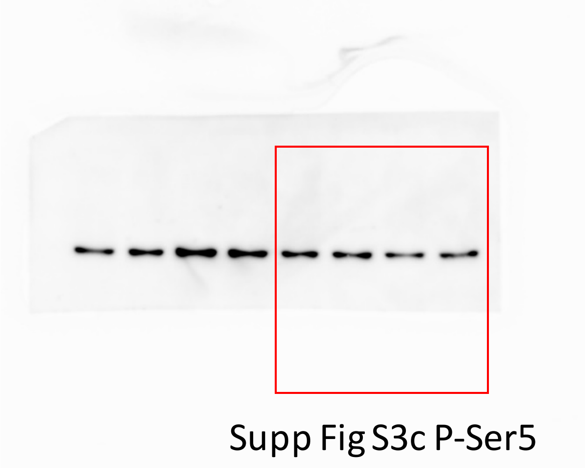

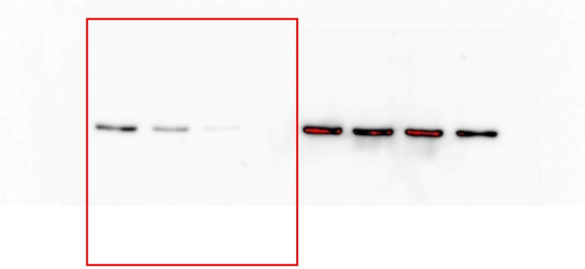

Supp Fig S3c P-Ser2
